# Comment on “Glasgow Coma Scale and Its Components on Admission: Are They Valuable Prognostic Tools in Acute Mixed Drug Poisoning?”

**DOI:** 10.1155/2011/159182

**Published:** 2011-10-31

**Authors:** Hossein Sanaei-Zadeh

**Affiliations:** Department of Forensic Medicine and Toxicology, Tehran University of Medical Sciences, Hazrat Rasoul Akram Hospital, Niayesh Street, Sattar-Khan Avenue, Tehran 1445613131, Iran

I read with interest the study performed by Eizadi Mood et al. recently published in Critical Care Research and Practice [1]. The authors investigated the value of Glasgow coma scale (GCS) on admission and its components (verbal, eye, and motor) in prediction of the outcome in mixed-drug poisonings. They found that the patients without complications had a greater mean value of GCS on admission and its components in comparison with those with complications. In “Material and Methods”, they explained that based on the patients' charts the outcomes were categorized as either without complications or with minor-to-severe (requiring intubation and ventilatory support) complications. 

Can they provide more details on the types of the complications they meant and their correlation to the GCS and its components? Also, may they provide the rate of intubation in these two groups (with or without complications)? In other words, did they only intubate the complicated patients or was there any uncomplicated patient who was intubated from the beginning and developed complications afterwards [2]? Thanks for this interesting paper.
